# Inner retinal oxygen metabolism in the 50/10 oxygen-induced retinopathy model

**DOI:** 10.1038/srep16752

**Published:** 2015-11-18

**Authors:** Brian T. Soetikno, Ji Yi, Ronil Shah, Wenzhong Liu, Patryk Purta, Hao F. Zhang, Amani A. Fawzi

**Affiliations:** 1 Functional Optical Imaging Laboratory, Department of Biomedical Engineering, Northwestern University, Evanston, IL; 2 Department of Ophthalmology, Northwestern University Feinberg School of Medicine, Chicago, IL; 3 Medical Scientist Training Program, Northwestern University Feinberg School of Medicine, Chicago, IL.

## Abstract

Retinopathy of prematurity (ROP) represents a major cause of childhood vision loss worldwide. The 50/10 oxygen-induced retinopathy (OIR) model mimics the findings of ROP, including peripheral vascular attenuation and neovascularization. The oxygen metabolism of the inner retina has not been previously explored in this model. Using visible-light optical coherence tomography (vis-OCT), we measured the oxygen saturation of hemoglobin and blood flow within inner retinal vessels, enabling us to compute the inner retinal oxygen delivery (irDO_2_) and metabolic rate of oxygen (irMRO_2_). We compared these measurements between age-matched room-air controls and rats with 50/10 OIR on postnatal day 18. To account for a 61% decrease in the irDO_2_ in the OIR group, we found an overall statistically significant decrease in retinal vascular density affecting the superficial and deep retinal vascular capillary networks in rats with OIR compared to controls. Furthermore, matching the reduced irDO_2_, we found a 59% decrease in irMRO_2_, which we correlated with a statistically significant reduction in retinal thickness in the OIR group, suggesting that the decreased irMRO_2_ was due to decreased neuronal oxygen utilization. By exploring these biological and metabolic changes in great detail, our study provides an improved understanding of the pathophysiology of OIR model.

Retinopathy of prematurity (ROP) is a major cause of blindness in children. In the United States alone, ROP affects approximately 550,000 preterm infants born each year (~11% of births)^1^, primarily premature infants with very low birth weight and gestational age^2^. A biphasic hypothesis has been proposed to explain the pathogenesis of the disease. At birth, the retinas of premature infants are incompletely developed and have an avascular peripheral zone^3^. In the first phase, hyperoxia occurs due to inhalation of either room air or supplemental oxygen, and suppresses hypoxia-induced vascular growth factors, leading to a delay in vascular maturation^4^. As the neuronal retina matures, its rate of oxygen consumption increases and outgrows the rate of oxygen delivery provided by its limited vascular supply, leading to hypoxia. In the second phase, retinal hypoxia results in the activation of hypoxia-inducible genes, which drive retinal angiogenesis and pre-retinal neovascularization^5^. Other biochemical mediators, including erythropoietin, growth hormone, and insulin-like growth factor-1, also appear to be important in mediating the neovascularization^3^,^6^. The newly formed, immature vessels lead to bleeding, fibrosis, and scarring. Eventually, if left unchecked, the fibrosis and scarring can evolve into retinal detachment and, ultimately, blindness^7^.

Rodent oxygen-induced retinopathy (OIR) models mimic the immature vascular development, oxygen susceptibility, and neovascularization that characterize ROP, and have contributed to an enhanced understanding of ROP pathogenesis. We chose to study the rat OIR model because it more closely mimics the exposure and retinal findings of human ROP than other models^2^. To induce 50/10 OIR in rats, Sprague-Dawley pups are exposed to alternating hyperoxia (50% O_2_) and hypoxia (10% O_2_) every 24 hours, beginning at birth and continuing until postnatal day 14 (P14)^8^. Upon return to room air at P14, the peripheral rat retina shows vaso-attenuation, which by P18, evolve into areas of peripheral neovascularization.

Although oxygen is thought to be a key player in the pathogenesis of OIR, ROP, and other proliferative retinopathies (e.g. diabetic retinopathy), oxygen metabolism has not been well studied in these diseases. In part, this may be due to the absence of imaging technologies than can quantify the inner retinal metabolic rate of oxygen (irMRO_2_) and inner retinal delivery rate of oxygen (irDO_2_), functional markers which fully describe the tissue oxygen consumption and oxygen delivery, respectively. Other markers, such as the oxygen saturation of hemoglobin (sO_2_), total retinal blood flow (F_Total_), and oxygen extraction fraction (OEF), contribute to the irMRO_2_ and irDO_2_; however, studying any of these measurements in isolation warrants caution because it provides a limited picture of oxygen metabolism. To illustrate how several of the biomarkers are connected, the irMRO_2_ equation can be written as follows:


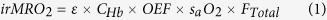


where 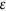 is the oxygen binding capacity of hemoglobin; 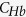 is the total hemoglobin concentration; and 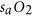 is the arterial oxygen saturation. A commonly used marker of oxygenation is the OEF, a unit-less quantity that represents the fraction of oxygen extracted from the blood and entering the tissue. The OEF can be written as follows:


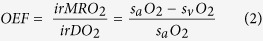


Note that, although irMRO_2_ and irDO_2_ individually depend on F_Total_, taking their ratio results in the cancellation of the flow terms; therefore, the OEF only depends on the oxygen saturation of hemoglobin for arteries (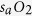) and veins (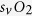). Since the OEF is a ratio, the interpretation of its value can be ambiguous. For example, an increase in the OEF can mean one of two things: either the irMRO_2_ is increased (increased oxygen consumption) or the irDO_2_ is decreased with the irMRO_2_ unchanged. Therefore, when interpreting the OEF, it is best to have measurements of irMRO_2_ and irDO_2_ to have a complete picture of oxygen metabolism.

Over the past 20 years, optical coherence tomography (OCT) has improved the diagnosis and management of retinal diseases by providing cross-sectional, high-resolution, structural imaging of the retina^9^. Recent advancements in OCT technology have steered the field towards functional imaging approaches. Using the Doppler effect, OCT can quantify retinal blood flow and perform retinal micro-angiography^10^,^11^,^12^,^13^. More recently, our group developed visible-light optical coherence tomography (vis-OCT), which can uniquely quantify sO_2_ in the inner retinal vessels *in vivo*^14^,^15^. Combining Doppler OCT, for measuring blood flow, with vis-OCT, for measuring oxygen saturation, enables the measurement of all the parameters required for irMRO_2_ and irDO_2_, in a single imaging device.

In this study, our primary objective was to use vis-OCT to quantify the irMRO_2_ and irDO_2_ in rats with 50/10 OIR. This is the first study that quantifies the irMRO_2_ in the 50/10 OIR model, providing important insight into retinal oxygen metabolism in proliferative retinopathies, including ROP.

## Results

### Multi-parameter assessment of oxygen delivery and metabolism with vis-OCT

**Figure 1:**
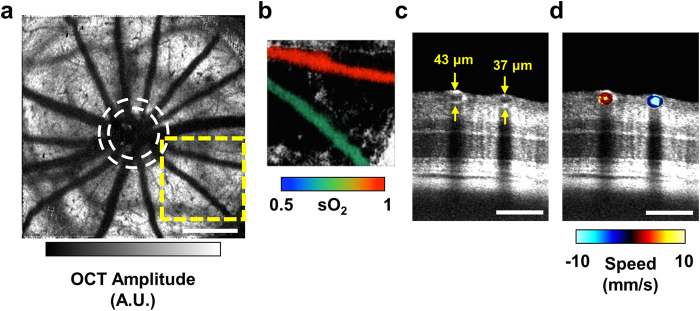
Multi-parameter assessment of oxygen delivery and metabolism with visible-OCT. (**a**) *En face* maximum amplitude projection of the shadows of the inner retinal vessels. White dashed circles indicate the approximate inner and outer scanning paths for dual-circle Doppler OCT. A.U.: arbitrary units. Scale bar: 500 μm. (**b**) An artery-vein pair from the yellow dashed box in (**a**) color-coded according to measured sO_2_. (**c**) An angular section from the outer circle of the dual-circle scan, showing the artery-vein pair in (**b**) and their respective measured diameters. Scale bar: 100 μm (**d**) The same artery-vein pair color-coded according to speed measured by Doppler OCT after phase unwrapping. Scale bar: 100 μm.

Using vis-OCT and dual-circle scanning Doppler OCT^11^,^14^,^16^, we obtained three-dimensional (3-D) anatomical images of the retina along with sO_2_, vessel diameter, and blood velocity. For each rat, we collected a series of two-dimensional B-scans by raster-scanning the illumination beam over an area of 2 × 2 mm^2^ and subsequently combined the series to form a 3-D image of the retina. Because hemoglobin is highly absorbing within the visible-light spectral range, shadows were cast underneath the inner retinal blood vessels. Taking advantage of this light attenuation, we selected a 3-D slab of retina below the inner retinal vessels and performed maximum amplitude projection (MAP), along the depth dimension, to obtain an *en face* image of the inner retinal vessel shadows. An example image of the shadows from a healthy rat at P18 is shown in Fig. 1(a). The contrast of the shadow image can be inverted and color-coded according to the measured sO_2_ values. Figure 1(b) shows an example of an artery-vein pair from Fig. 1(a), carrying oxygenated (97%) and deoxygenated (71%) blood to and from the retinal periphery, respectively.

To measure vessel diameter and blood velocity, we implemented a dual-circle scanning protocol on the same vis-OCT system^11^,^17^. The dual-circle scanning method does not require an entire 3D volumetric scan. Instead, the illumination beam is rapidly scanned around the optic disk in two concentric circles such that all the major blood vessels are captured, as depicted by the dashed circles in Fig. 1(a). Figure 1(c) shows an angular section from the outer circle scan, which matches the artery-vein pair shown in Fig. 1(b). An example of blood velocity measurements for the pair is shown in Fig. 1(d).

The accuracy of sO_2_ and blood velocity measurement is supported by previous studies from our group. Using vis-OCT, Yi *et al*. monitored arterial sO_2_ in a mouse ear in response to hyperoxia and hypoxia challenges^18^. The study found high correlation between vis-OCT measurements and pulse-oximetry readings. In another study, Song *et al*. confirmed that a 25 kHz sampling rate and averaging measurements over multiple scans enabled accurate mean blood flow measurements in rodents using the dual-circle scanning Doppler OCT^16^.

### Inner retinal sO_2_, diameter, blood velocity, and blood flow

**Figure 2:**
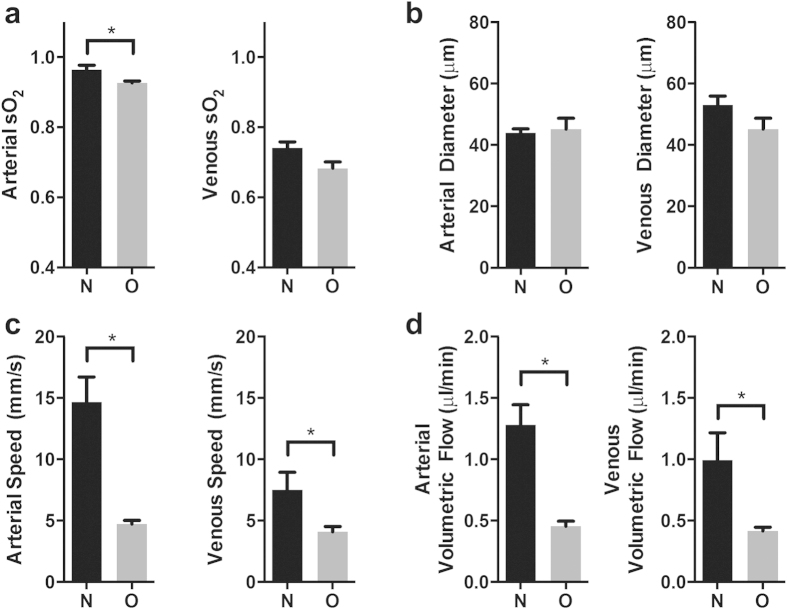
Comparison of retinal metabolic parameters between room-air-raised and oxygen induced retinopathy (OIR) rats at P18. (**a**) Average arterial and venous sO_2_ measurements acquired with vis-OCT in room-air-raised (N; n = 6) and OIR rats (O; n = 4). (**b**) Arterial and venous diameter measurements acquired with from dual-circle scanning OCT. (**c**) Arterial and venous speed measurements acquired with Doppler OCT. (**d**) Calculated arterial and venous volumetric flow for the two groups. *p < 0.05.

Figure 2(a) shows a comparison of arterial and venous sO_2_ measurements between room-air-raised rats (N; n = 6) and rats with OIR (O; n = 4) at P18. In the control animals, the average arterial sO_2_ was higher when compared with the arterial sO_2_ in the OIR group (0.964 ± 0.013 vs. 0.928 ± 0.006; p < 0.05). This finding may be explained by the physiologic differences between the two groups. In particular, rats with 50/10 OIR have poor weight gain and decreased systemic arterial oxygen (similar to values seen in infants in the neonatal ICU) when compared with their room-air-raised controls^19^. A statistically significant difference was not found between the average venous sO_2_ in the N and O groups (0.741 ± 0.017 vs. 0.684 ± 0.023; p = 0.09).

Our sO_2_ results for the control rats agreed with previous investigations of the inner retina sO_2_ in normal rats. Yi *et al*. used vis-OCT to measure inner retinal arterial and venous sO_2_ in Long Evans pigmented rats and found the sO_2_ to be 95% and 72%, respectively^14^. Song *et al*. used photoacoustic ophthalmoscopy (PAOM) to image normal adult Sprague-Dawley rat and obtained an average arterial and venous sO_2_ of 93.0% and 77.3%, respectively, which match well with our measurements using vis-OCT^16^. In another study, using phosphorescence lifetime measurements to measure intravascular partial pressure of oxygen (pO_2_) in Long Evans pigmented rats, the authors found arterial sO_2_ and venous sO_2_ (after conversion from pO_2_ using the hemoglobin disassociation curve) in normal conditions to be 61% and 34%, respectively. These findings were lower than those in our study; however, the authors noted that their measurements were lower than those seen in humans and that anesthesia may have induced respiratory depression, leading to systemic hypoxia^20^.

Figure 2(b) compares the arterial and venous diameter between the N and O groups. Arterial diameter was not significantly different between controls and rats with OIR (43.9 ± 1.4 μm vs. 45.2 ± 2.9 μm; p = 0.72). Likewise, venous diameter was not significantly different between controls and rats with OIR (53.1 ± 2.9 μm vs. 48.1 ± 2.5 μm; p = 0.23). Our diameter measurements of the major arteries and veins in the OIR group were relatively similar to those reported using *ex vivo* retinal flatmounts for arteries (45.1 μm) and veins (45.7 μm) in this model^8^. Figure 2(c) shows the average arterial and venous blood flow speeds for the two groups. Arterial blood flow speed was significantly higher in controls compared to rats with OIR (14.68 ± 2.03 mm/s vs. 4.65 ± 0.37 mm/s; p < 0.01). Similarly, venous blood flow speed was also significantly higher in controls compared to rats with OIR (7.52 ± 1.41 mm/s vs. 3.71 ± 0.31 mm/s; p < 0.05). Figure 2(d) shows the average arterial and venous volumetric flows. Average arterial volumetric flow was significantly different between control and rats with OIR (1.28 ± 0.16 μl/min vs. 0.45 ± 0.05 μl/min; p < 0.01). Similarly, average venous volumetric flow was significantly different between control and rats with OIR (−1.00 ± 0.40 μl/min vs. 0.59 ± 0.06 μl/min; p < 0.05). Overall, we successfully obtained high quality flow measurements from 70% of the vessels in the control rats and 50% of the vessels in rats with OIR. There was no significant difference in the number of major inner retinal vessels between controls and rats with OIR (13 ± 0.45 vessels vs. 13 ± 1.3 vessels; p > 0.99). Across all measurements in the control group, the average Doppler angle was 101 ± 3 degrees. In the rats with OIR, the average Doppler angle was 102 ± 3 degrees. All measurements had Doppler angles greater than 95 degrees.

### Total retinal blood flow, oxygen delivery, oxygen extraction fraction, and metabolic rate of oxygen

**Figure 3:**
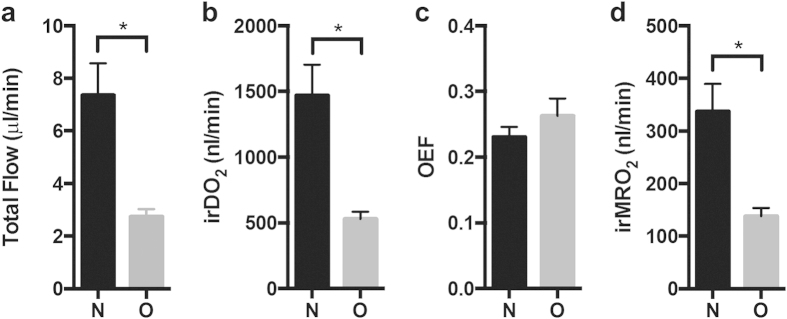
Inner retinal oxygen delivery (irDO_2_) and metabolic rate of oxygen (irMRO_2_) are decreased in OIR rats at P18. (**a**) Estimated total blood flow (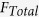) for room-air-raised controls (N; n = 6) and rats with OIR (O; n = 4) (**b**) inner retinal oxygen delivery (irDO_2_) measurements for N and O groups. (**c**) Oxygen extraction fraction (OEF) measurements for the N and O groups. (**d**) Inner retinal metabolic rate of oxygen (irMRO_2_) measurements for N and O groups. *p < 0.05.

As shown in Fig. 3(a), the average F_Total_ in rats with OIR was significantly lower than in controls (2.74 ± 0.58 μl/min vs. 7.37 ± 2.96 μl/min; p < 0.05). F_Total_ measurements for the controls were consistent with previously reported measurements made using *en face* Doppler OCT in adult Sprague-Dawley rats (6.48 μl/min)^21^, and fluorescent microsphere imaging in adult Long Evans pigmented rats (7.9 μl/min)^22^,^23^. We found significant reduction in irDO_2_ in rats with OIR versus controls as shown in Fig. 3(b) (568.0 ± 54.99 nl min^−1^ vs. 1473 ± 231.9 nl min^−1^; p < 0.05).

Because the OEF calculation does not require blood flow measurements, the OEF is sometimes used to characterize the oxygen metabolism in tissue. OEF is described only by sO_2_ values (see Equation 2); therefore, it only serves as a static measure of oxygen metabolism. As shown in Fig. 3(c), there was no significant difference in the OEF between the two groups (0.231 ± 0.037 vs. 0.262 ± 0.054; p = 0.36). In comparison, Fig. 3(d) shows that irMRO_2_ was significantly lower in rats with OIR versus controls (138 ± 16 nl min^−1^ vs. 338 ± 52 nl min^−1^; p < 0.05). As we discussed in the introduction, the OEF is a unit-less ratio, which can sometimes be difficult to interpret. Case in point, we found that the OEF was not significantly different when comparing normal rats to rats with OIR. This can be explained by the fact that the irMRO_2_ and the irDO_2_ were proportionately decreased. We could only determine the latter because we measured both F_Total_ and sO_2_. Our study, therefore, cautions scientists to avoid drawing conclusions based on OEF measurements alone, without considering blood flow and metabolic rate of oxygen.

Our irMRO_2_ measurements in normal rats agree with those obtained using a combination of PAOM and Doppler OCT in adult Sprague Dawley rats (297.86 nl O_2_/min)^24^. However, using phosphorescence lifetime imaging to measure pO_2_ and fluorescent microsphere imaging to measure F_Total_, in two separate studies, Wanek *et al*. measured the irMRO_2_ to be ~500 nl O_2_/min in normal adult Long Evans pigmented rats, which is higher than what we obtained in our current study^22^,^23^. These differences in the normal irMRO_2_ could be attributed to species and age differences between the animals in the respective studies. Notably, differences in the anesthesia protocol are known to affect F_Total_, which could account for differences in the calculated irMRO_2_. In particular, due to its vasodilating action, isofluorane anesthesia may result in higher flow speeds than ketamine/xylazine used in our study^10^.

### Retinal avascularity, clock hours with neovascularization, and retinal vascular density

**Figure 4:**
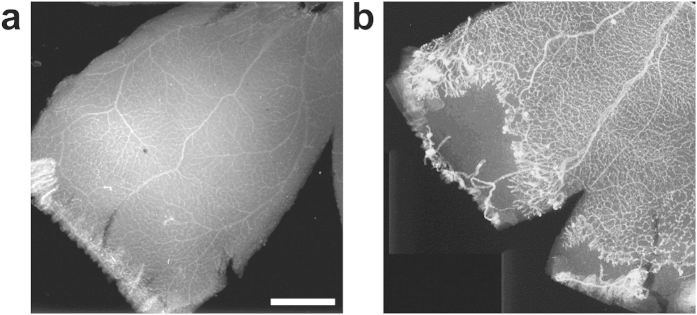
Immuno-stained retinal flatmounts show peripheral avascular retina and neovascular tufts in OIR rats at P18. (**a**) Room-air-raised control. (**b**) Rat with oxygen-induced retinopathy OIR. Scale bar: 1 mm.

To investigate the reason for decreased irDO_2_, we performed fluorescence microscopy on isolectin-stained flatmounts of the retinal vasculature. Figure 4(a,b) show representative flatmounts from controls and rats with OIR. The rats with OIR (n = 4) had 4.25 ± 2.25 clock hours with neovascularization. Rats with OIR had on average 10.92 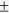 3.50% peripheral retinal avascular areas. By definition, normal rat eyes had completely vascularized peripheral retina and zero clock hours of neovascularization. In this study, the number of clock hours with neovascularization was similar to that reported at P19 (~3.5 clock hours)^25^, but was less than that originally reported by Penn *et al*. at P18 (8 clock hours)^8^. Our measurements of peripheral avascularity measured in this model were less than those reported by Akula *et al*. (~20%) and Penn *et al*. (25.2% peripherally and 4.2% centrally)^8^,^25^.

**Figure 5:**
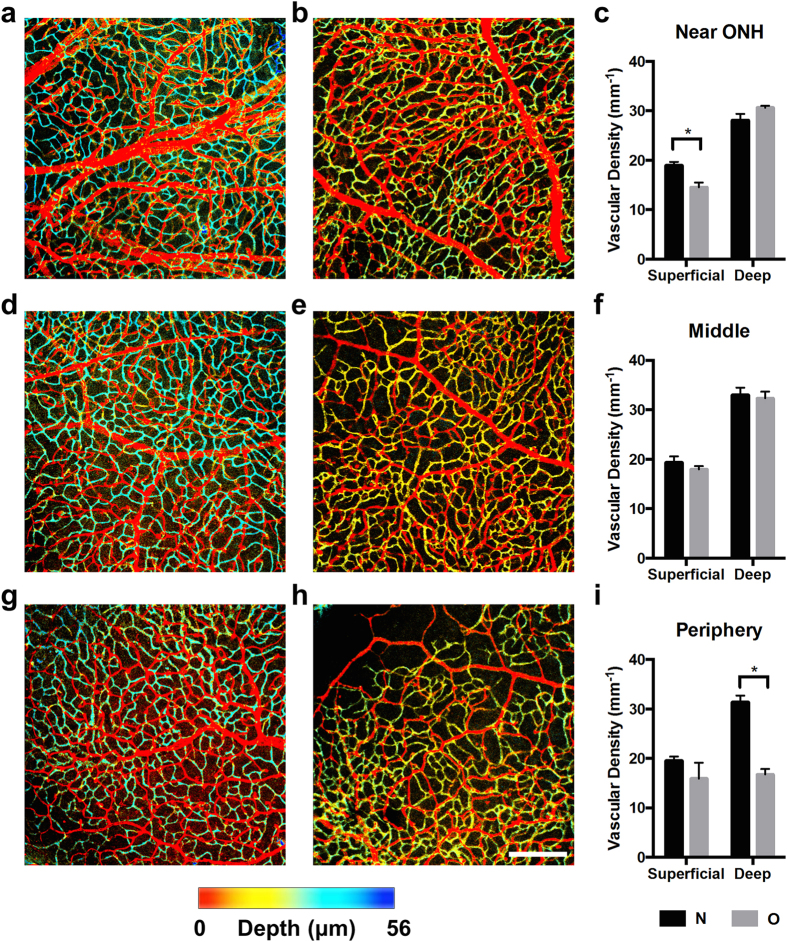
Retinal vascular density of the superficial and deep vascular networks is decreased in the OIR rats. (**a**) *En face* maximum amplitude projection of the vasculature near the optic nerve hear (ONH) acquired with confocal microscopy in a healthy rat at P18, which has been color-coded according to depth. (**b**) Vasculature near the ONH in a rat with OIR. (**c**) Vascular density measurements near the ONH. (**d**) Vasculature at halfway between the ONH and the periphery (middle) in a room-air-raised control. (**e**) Vasculature at halfway between the ONH and the periphery and in a rat with OIR. (**f**) Vascular density measurements in the middle. (**g**) Vasculature at the periphery in a room-air-raised control. (**h**) Vasculature at the periphery in a rat with OIR. Scale bar: 200 μm. (**i**) Vascular density measurements at the periphery. *p < 0.05.

To study the vascular density in the superficial and deep capillary plexi, we used confocal microscopy to optically section the isolectin-stained flatmounts. We imaged the retinal flatmounts at three locations: near the optic nerve head (ONH), halfway between the ONH and the periphery (middle), and near the periphery. At those three locations, we measured the vascular density in room-air-raised rats (N; n = 4) and rats with OIR (O; n = 4). Figure 5(a) shows a representative image of the vasculature near the ONH in room-air-raised rats. The image was color-coded according to depth; therefore, the superficial vascular network was colored red, while the deep capillary network was colored blue. Figure 5(b) shows a representative image of the vasculature near the ONH in a rat with 50/10 OIR. Here the deep capillary network is colored yellow-green because the retina was thinner in this group (see next section), and thus, the deep capillary network was closer to the superficial vessels. Figure (c) compares the measured vascular density for the N and O groups. We found that superficial vascular density in the O group was significantly decreased near the ONH compared to the N group (14.5 ± 1.0 mm^−1^ vs. 19.0 ± 0.7 mm^−1^; p < 0.05), but differences in deep vascular density were not statistically significant (30.7 ± 0.4 mm^−1^ vs. 28.1 ± 1.2 mm^−1^; p = 0.14).

Figure 5(d,e) show representative images of the vasculature taken halfway between the ONH and the periphery, and Fig. 5(f) shows the corresponding vascular density measurements. We did not find statistically significant differences in superficial or deep vascular density between the N and O groups at this location (superficial: 19.3 ± 1.2 mm^−1^ vs. 18.0 ± 0.6 mm^−1^; p = 0.37; deep: 33.0 ± 1.5 mm^−1^ vs. 32.3 ± 1.4 mm^−1^; p = 0.74). Figure 5(g,h) show representative images of the vasculature at the periphery, and Fig. 5(i) shows the corresponding vascular density measurements. We found no statistically significant difference in the superficial vascular density (19.5 ± 0.8 mm^−1^ vs. 15.9 ± 3.2 mm^−1^; p = 0.35); however, the O group showed a statistically significant decrease in deep capillary density (31.4 ± 1.3 mm^−1^ vs. 16.7 ± 1.1 mm^−1^; p < 0.001). These results reflect observations that the superficial vascular network develops before the deep capillary network in rats^26^.

In a previous study, Wang *et al*. found that vascular density in the superficial and deep networks was decreased in 50/10 OIR rats compared with room-air-raised controls^27^. Using wide-field fluorescence microscopy, the authors measured a single vascular density for the entire retina. In this study, we measured the density in distinct locations across the retina using confocal microscopy. Our results suggest that differences were located in the superficial network near the ONH, while the effects on the deep vascular network were more prominent in the retinal periphery.

### Retinal layer thickness

**Figure 6:**
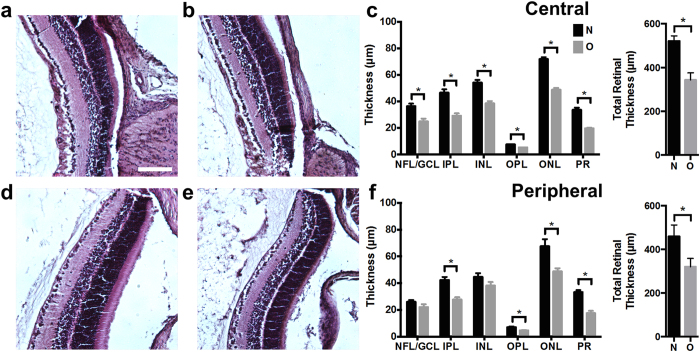
Retinal sublayer thinning in rats with OIR at P18. (**a**) Example central histological section from a room-air-raised control. Scale bar: 100 μm. (**b**) Example central histological section from a rat with OIR. (**c**) Central retinal sublayer thickness and total central retinal thickness measurements from room-air-raised controls (N; n = 4) and rats with OIR (O; n = 4). (**d**) Example peripheral histological section from a room-air-raised control. (**e**) Example peripheral histological section from a rat with OIR. (**f**) Peripheral retinal sublayer thickness and total peripheral retinal thickness measurements. *p < 0.05. Layer name abbreviations: Nerve fiber layer and ganglion cell layer (NFL/GCL), inner plexiform layer (IPL), inner nuclear layer (INL), outer plexiform layer (OPL), outer nuclear layer, (ONL), photoreceptor layer (PR).

To investigate the reason for decreased oxygen consumption in rats with OIR, we performed histologic cross-sections to measure retinal sublayer thicknesses. Representative histological sections of the central retina for a normal rat and rats with OIR are shown in Fig. 6(a,b) respectively. As shown in Fig. 6(c), we found that all retinal layers in the central retina were significantly thinner in rats with OIR (O; n = 4) than controls (N; n = 4) (p < 0.05). Figure 6(d,e) show representative peripheral retinal sections for a healthy rat and a rat with OIR respectively. We found that the OIR peripheral retina had statistically significant thinning of the inner plexiform (IPL), outer plexiform (OPL), outer nuclear (ONL), and photoreceptor layer (PR). These measurements were highly correlated between the two masked graders (Pearson’s correlation coefficient > 0.85 for all layers except the OPL which had a correlation coefficient of = 0.53).

Our total retinal thickness measurements in room-air-raised rats (central: 242 μm; peripheral 160 μm) were similar to those made by Akula *et al*. at P19 (central: 225 μm; peripheral: 205 μm)^25^. In addition, our total thickness measurements in 50/10 OIR (central: 213.5; peripheral 149.0 μm) were also similar to thickness measurements in rats with 50/10 OIR at P19 (central: 185 μm; peripheral: 164 μm). In their study, Akula *et al*. estimated approximately a 40% decrease in retinal volume in the rats with OIR. Similar to our results, these authors also concluded that the post-receptor layers were significantly thinner in rats with 50/10 OIR than in controls.

## Discussion

We found a significant reduction in inner retinal oxygen delivery in rats with OIR compared to controls at P18 (Fig. 3(b)). Decreased F_Total_ contributed the most to the reduced oxygen delivery (Fig. 3(a)); however, the reason for the reduced F_Total_ was not readily apparent. The Hagen-Poiseuille law relates volumetric flow, 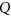, and pressure drop, 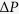, across a single cylindrical pipe, given by the following equation:


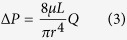


where 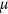 is the blood viscosity, 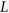 is the length of the tube, and 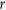 is the vessel radius^28^. According to this law, reductions in the radii of the major retinal vessels could significantly affect the flow magnitude. However, we found that diameter measurements obtained *in vivo* in major arteries and veins were not significantly different between controls and rats with OIR (Fig. 2(b)). Therefore, the difference in F_Total_ between the two experimental groups does not arise from changes in the vessel caliber at the pre-capillary or post-capillary level in the inner retinal vascular network.

Alternatively, the magnitude of 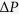 could significantly affect F_Total_. The pressure drop across an inner retinal vessel depends on the systemic arterial and venous pressures as well as the intermediate resistance of the capillary bed. The former is less likely to be different in rats with OIR compared to controls; therefore, we hypothesized that increased capillary bed resistance in rats with OIR was leading to decreased blood velocity in rats with OIR, as shown in Fig. 2(c), and thereby, decreased F_Total_. Similar findings have been observed in patients with hypertension and reduced retinal blood velocity^29^.

A number of possibilities potentially explain why the capillary bed may have high resistance. Presumably, the peripheral neovascular lesions could contribute to increased vascular resistance, especially considering how this is one of the more striking vascular findings at P18; however, this is unlikely because we found that the tufts lay only in the periphery and occupied a small percentage of the retinal area (4 clock hours). Instead, we hypothesized that the overall retinal capillary bed was abnormal in rats with OIR at P18. Plausible structural abnormalities, which could significantly increase resistance, included either increased dynamic capillary closure or an overall decrease in the vascular density. To investigate the latter possibility, we measured vascular density in isolectin-stained retinal flat mounts imaged with high-resolution confocal microscopy (Fig. 5). In rats with OIR at P18, we observed decreased capillary density in the superficial capillary bed near the ONH and in the deep capillary bed in the periphery. These findings suggest that oxygen delivery is reduced at P18 in rats with OIR because of these differences in the construction of the vascular tree; however, they do not exclude the possibility that there is potential dynamic capillary closure in addition to the changes in the overall vascular density. Future *in vivo* studies using micro-angiography techniques may be able to assess whether capillary closure is a contributing factor.

The findings of decreased oxygen delivery appear to be consistent with functional magnetic resonance imaging (MRI) measurements of functional oxygenation, revealed by measurement of the change in pO_2_ (ΔpO_2_) in response to an oxygenation challenge in rats with 50/10 OIR. Berkowitz *et al*. found a lower ΔpO_2_ in response to a 2-minute carbogen breathing challenge in OIR, when compared to controls at P12 (before the appearance of neovascularization)^30^. A similar significant lowering of the ΔpO_2_ at P20 (after neovascularization) was also found^31^. The authors cited poor retinal perfusion, which would limit oxygen delivery, as one potential explanation for the decreased ΔpO_2_; however, blood flow measurements were not conducted to verify this possibility. The decreased oxygen delivery and decreased vascular density found in our study could explain the abnormal ΔpO_2_ in these studies. In addition, the decreased oxygen delivery would also serve as an explanation why Saito *et al*. found that supplemental oxygen did not reduce concentrations of conjugated retinal pimonidazole, a marker of retinal hypoxia, at P18^32^. Supplemental oxygen would likely not be able to overcome the significant decrease in total retinal blood flow and vascular density.

Given the decreased oxygen delivery found at P18, we expected the OEF to be elevated in rats with OIR. In states of ischemia (i.e. decreased oxygen delivery), the metabolic oxygen consumption of the tissue is usually elevated relative to the oxygen delivered, and thus a greater percentage of hemoglobin-bound oxygen diffuses through the capillary wall to compensate, resulting in an increased OEF measurement. This type of pathophysiology – termed “misery perfusion” in the field of neurology – is commonly seen in the setting of acute ischemic stroke^33^. Surprisingly, however, we found only a modest elevation in OEF (Fig. 3(c)). The minimal increase in OEF at P18 in the OIR rats suggested that although there was diminished oxygen delivery, the oxygen consumption of the retina was also reduced. Indeed, irMRO_2_, which reflects the oxygen consumption of the retina per unit time, was reduced, proportionate to the decrease in oxygen delivery (Fig. 3(d)). To explain this unexpected decrease in irMRO_2_, we hypothesized that there was an overall decrease in the number of cells and synapses and, therefore, a lower inner retinal oxygen demand in OIR rats. To investigate this hypothesis, we compared retinal sublayer thicknesses in control and OIR eyes (Fig. 6(a,b,d,e)), and confirmed that the retinal layers were significantly thinner in rats with OIR (Fig. 6(c,f)). Previous electroretinography studies further support our observation^34^; receptor sensitivity, postreceptor sensitivity, and oscillatory potentials were reduced at P20 in OIR rats when compared with normal controls^35^,^36^. In summary, irMRO_2_ at P18 in OIR rats is markedly reduced, likely owing to decreased retinal thickness and decreased oxygen demand throughout the retinal layers.

The OEF and irMRO_2_ findings at P18 potentially explain some of the peculiar characteristics of the retinopathy in rats with OIR. Interestingly, isolectin-stained flatmounts at P30 in rats with 50/10 OIR show a fully vascularized retina with no signs of neovascularization^25^. In other words, at some point between P18 and P30, the neovascularization regresses and the retinal periphery becomes completely vascularized. How can we extrapolate from our results to explain this phenomenon ? After exiting the oxygen chamber at P14, the oxygen consumption of the retina most likely exceeds the oxygen delivery, resulting in a state of hypoxia and, presumably, high OEF. Supporting this statement, several studies have observed that VEGF levels appear to be the highest when returning to room air at P14 in the 50/10 model^37^,^38^,^39^. Through VEGF, hypoxia at P14 likely drives the neovascularization, which is maximally observed at P18. Further exacerbating this hypoxia, vaso-attenuation of the peripheral vasculature also may progress between P14 and P18 as one study found^25^; however, another study on rats with OIR found the contrary^38^, so it is currently unclear if this plays a significant role. Around this same time period in normal neonatal rat eye development (from P10 to P25), the photoreceptors and retinal neurons undergo physiologic culling, whereby an initial excess of photoreceptors undergo apoptosis to reach levels for adult life. Hypoxia has been shown to substantially exacerbate this process, especially from P15 to P22 in rats^40^,^41^. Therefore, at P14 in OIR, the simultaneous occurrence of hypoxia and physiologic culling likely causes a dramatic increase in neuronal apoptosis, which in turn decreases the overall oxygen demand of the retina. Our retinal thickness, OEF, and irMRO_2_ results suggest that by P18 in OIR, the oxygen demand has sufficiently decreased to almost match the pathologically decreased oxygen delivery. Beyond P18, the neuronal culling continues, and oxygen delivery and consumption become progressively balanced, decreasing the stimulus for angiogenesis. Eventually, the neovascularization subsides, and the physiologic retinal vascular development proceeds. Further studies are necessary to confirm this hypothesis, but our study is the first to suggest this as a likely possibility.

We note several limitations to this study. First, the equation used to calculate irMRO_2_ in this study is only an approximation to the true irMRO_2_ equation. To model oxygen delivery physically, the irMRO_2_ equation should include individual feeding and draining vessels as separate terms instead of using averaged quantities for flow and oxygen saturation^42^. This latter equation has not been demonstrated in the retina as of yet likely because it requires measurement of all feeding and draining vessels and highly accurate flow speeds to ensure that feeding and draining volumetric flows are closely conserved. In this study, a combination of hyaloid vasculature, vitreous hemorrhage, and cataract formation, which were more common in rats with OIR, limited our ability to obtain high quality flow speeds and sO_2_ measurements for all the retinal vessels, and precluded the use of a more specific irMRO_2_ equation. To ensure accurate flow measurement, we selected vessels with high signal-to-noise ratio and made averages over 16 scans to improve imaging quality. Second, our irMRO_2_ equation does not take into account pO_2_. However, owing to low solubility of oxygen in blood, intravascular pO_2_ only makes up a small percentage (<2%) of the oxygen carried in the blood, and thus it is reasonable to disregard pO_2_^43^. Third, other factors may contribute to the decreased F_Total_ besides capillary bed resistance and vessel diameter. These factors include the cardiac output, total circulating blood volume, ocular pressure, and weight gain of the subjects, which were not measured in this study. Finally, vessel diameters were measured by fitting an ellipsoid over the hyper-reflective vessel walls, which is likely to be greater than the true inner diameter of the vessel; therefore, our diameter measurements may overestimate the F_Total_. However, since such an overestimation was systematic, comparisons between groups should not be affected because diameters were measured consistently.

Future studies for longitudinal measurement of the irMRO_2_ with vis-OCT would further elucidate the pathophysiology of the OIR model; however, several challenges remain. First, this OIR model relies upon the tightly regulated oxygen control within the animal chamber from P0 to P14; therefore, prolonged exposure to room air for imaging experiments before P14 would significantly disrupt the animal model. Second, imaging experiments at P14 may prove difficult given the significant persistence of the hyaloidal vasculature and developmental eyelid closure, which opens around P14-15 in the rat. Finally, at P18 and beyond, cataract and vitreous opacity in the OIR model may also hinder satisfactory image quality.

In summary, we capitalized on the unique capability of vis-OCT to simultaneously measure sO_2_, vessel diameter, and blood flow, and quantified the retinal MRO_2_ in healthy neonatal rats and OIR rats, for the first time. In addition, we combined this new technology with immunostaining and histological analysis to gain an improved understanding of the biological implications of these metabolic measurements. The findings in this study help explain the pathophysiology of oxygen consumption and delivery in rats with OIR, which can be extrapolated to retinal diseases involving inner retinal hypoxia and retinal angiogenesis in humans, including diabetic retinopathy. Our findings lay the foundation for future studies to explore retinal oxygen metabolism at various time-points in the progression of retinopathy in this animal model, as well as human studies of ROP and diabetic retinopathy at various stages of their development. We envision that future technological improvements to vis-OCT, with enhanced speed and scan range, will permit volumetric structural measurements of the entire retina (ora-to-ora), and improved segmentation of the retinal sublayers, which would allow more detailed correlation between structural alterations and retinal oxygen consumption.

## Methods

### Animal model

All animal studies were approved and performed in compliance by the Institutional Animal Care and Use Committee at Northwestern University and the Association for Research in Vision and Ophthalmology States for the Use of Animals in Ophthalmic and Vision Research. The 50/10 rat OIR model has been previously described^8^. Within six hours after birth, Sprague-Dawley (Charles River, Wilmington MA) rat pups and their nursing moms were placed in a Plexiglas chamber with an oxygen controller (Pro-Ox 110; Biospherix, Lacona, NY). Starting on P0, the oxygen concentration was cycled between 50% and 10% every 24 hours for 14 days. After 14 days, the pups were returned to room air. Age-matched control pups were maintained in room air throughout the duration of the experiment. At P18, the OIR and control pups were imaged using vis-OCT and, following imaging, were euthanized for retinal harvest.

### Animal preparation

The anesthesia protocol used during the vis-OCT imaging session was 0.25–0.3 ml ketamine/xylazine mixture (ketamine: 11.45 mg/ml; xylazine:1.7 mg/ml, in saline), injected intraperitoneally. The body temperature of the animal was maintained at 37 °C using a heating pad (homeothermic blanket system, Stoelting Co.). We applied 0.5% tetracaine HCl ophthalmic solution to the eyes for local anesthesia and 1% tropicamide ophthalmic solution to dilate the pupil. Commercial artificial tears were applied to the eyes during imaging to prevent corneal dehydration. The pups were maintained on a custom-made animal holder during the imaging session.

### Quantification of sO_2_

Measurements of sO_2_ were made using our vis-OCT system described previously^14^. Briefly, vis-OCT utilizes the prototypical frequency-domain OCT optical design based on the Michelson interferometer. Instead of using a broadband laser source in the infrared spectrum, vis-OCT incorporates a supercontinuum source (NKT photonics, SuperK), which has a spectrum from 500 nm to 620 nm. The sO_2_ was determined as follows. First, to segment the inner retinal vessels from the background, we utilized the contrast between the background and the retinal vessel shadows and applied Otsu’s method to automatically determine a global threshold for vessel segmentation^44^. Once the inner retinal vessels were segmented and manually selected, the vessel centerlines were determined. Since OCT system uses broadband light source, spectroscopic analysis is possible at any given location^45^. Thus, short-time Fourier transforms were calculated at the centerline to extract the spectra from the bottom of the vessel wall. Based on the distinct absorption spectrum from oxygenated and deoxygenated hemoglobin, the sO_2_ can be calculated by fitting the vis-OCT spectrum with the standard hemoglobin spectra^14^,^18^. All post-processing was performed in MATLAB (R2012b, MathWorks).

### Quantification of retinal blood vessel diameter, blood velocity, and blood flow

Assuming the rat eye diameter is approximately 3 mm at P18^25^, the estimated inner and outer radius of the circular scans across the retina were 0.21 mm and 0.31 mm, respectively. The difference in displacement of the blood vessels between the two circular B-scans can be used to calculate the Doppler angle (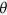) – the angle between the illumination beam and the flowing blood. Equations for calculating the Doppler angle with this setup can be found in the supplementary section of a previous study by Song *et al*.^16^. To obtain valid velocity measurements, we aimed to keep the Doppler angles approximately 10 degrees away from 90 degrees (i.e. 80 or 100 degrees). The phase component of the inverse Fourier transform of the OCT interferometric signal was then used in combination with the Doppler angle to calculate the flow speed. The phase difference between adjacent A-lines after bulk-motion correction, 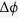, is proportional to the flow speed^46^. The blood flow speed can be calculated with 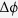 and 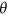 with the equation:


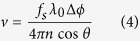


where f_s_ is the A-line scanning frequency, λ_0_ is the center wavelength of the light source, and n is the refractive index of the sample (f_s_ was 25 kHz; λ_0_ was 568 nm; and n was 1.4)^11^. For each vessel, 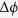 was averaged over 16 scans to obtain an average velocity. To correct for phase wrapping, the blood flow direction was inferred from the velocity at the vessel boundary, where flow is slow enough to not suffer from phase-wrapping. Additionally, using the sO_2_ measurements obtained with vis-OCT, each vessel was labeled as an artery or a vein, which also assisted in the determination of blood flow direction (arteries flow away from the optic nerve towards the periphery and veins vice versa).

To determine the vessel diameter, 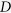, ellipsoids were manually user-fitted in MATLAB over the hyper-reflective vessel wall seen in the OCT structural images obtained from the inner and outer circular scans. Because the depth resolution (1.07 μm) was much higher than the transverse resolution (6 μm), only the depth dimension was used to measure the vessel diameter, as depicted by the arrows in Fig. 1(c). The diameter measurements were then averaged across all scans. Assuming cylindrical vessels, the cross-sectional area is given by 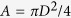. Assuming laminar flow within the blood vessels, the product of the cross sectional area, A, and average blood velocity, 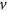, yields the volumetric blood flow, F^47^.

### F_Total_, irDO_2_, OEF, and irMRO_2_ quantification

F_Total_ (in ml blood/min) was calculated using the equation:


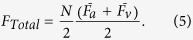



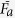
 and 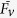 are the average arterial and venous blood flows, respectively. 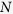 indicates the total number of vessels counted on the *en face* shadow image.

Inner retinal oxygen delivery, irDO_2_, was calculated using the following equation:


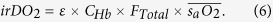


Here 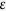 is the oxygen binding capacity of hemoglobin (1.36 ml O_2_/g of Hb)^48^. 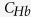 is the total hemoglobin concentration (150 g of Hb/L of blood). OEF was calculated using the following equation:


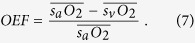


where 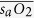 and 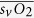 are the average arterial and venous sO_2_ percentages, respectively. irMRO_2_ was calculated based on total blood flow and arteriovenous sO_2_ differences using the equation:


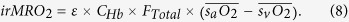


which is equivalent to Equation 1.

The units of irDO_2_ and irMRO_2_ were in nl min^−1^.

### Retinal dissection, flat mounts, and immunostaining

Rat eyes were enucleated and fixed for 24 hours in 10% neutral buffered formalin at room temperature and then transferred to 70% EtOH. The retinal eye cups were dissected as described previously^49^. The retinas were washed with phosphate buffer saline (PBS) for three hours before being placed overnight in PBS with 0.5% Triton X-100 solution to help improve vascular visibility. The following day the retinas were washed in PBS for three hours and then stained overnight in 4 °C with Alexa Fluor 594 isolectin GS-IB4 conjugate (1:75 dilution; Invitrogen, Carlsbad, CA) in PBS with MgCl_2_, CaCl_2_, and 0.3% Triton X-100 solution. Following a two hour wash in PBS with 0.025% Triton X-100 solution, the retinal cups were cut in quadrants, flat mounted, and cover slipped with ProLong Gold mounting medium (Life Technologies, Carlsbad, CA, USA).

Single-plane fluorescence images of the immunostained retinal flatmount were acquired with Nikon Eclipse 80i upright microscope (Nikon Instruments Inc, Melville, NY, USA) using a Photometrics ES CoolSnap camera (Photometrics, Tuscan, AZ, USA) and MetaMorph software (Molecular Devices, Sunnyvale, CA, USA). Five individual images of the retinal flaps were taken at 2× magnification and then merged using the stitching plugin in ImageJ software (National Institutes of Health, Bethesda MD)^50^,^51^. For each eye, two masked graders (A.A.F. and P.P.) independently measured the avascular area and neovascularization, and the average of their measurements was used for subsequent analysis. To measure avascular area, graders traced both avascular and total retinal areas using the “Freehand selections tool” in ImageJ. Avascularity was computed as the ratio of the avascular to the total retinal area. To score neovascularization, each retinal quadrant was divided into three equal parts (clock hour) and the total number of clock-hours with neovascularization was counted as described previously^52^.

To examine the vascular density of the superficial and deep networks, we used confocal microscopy (543 nm, Zeiss LSM 510, Jena, Germany) to image the isolectin-stained retinal flatmounts. Two z-stacks per eye were obtained near the optic nerve head, halfway between the optic nerve head and periphery, and at the periphery of the retinal flaps. The dimensions of the z-stacks were 900 μm × 900 μm × 120 μm with a z-interval of 1.4 μm, resulting in approximately 85 images (depending on the retinal thickness). To avoid measurement error of the vascular density, images were captured in areas without any immunostaining artifacts. In addition, the two z-stacks were captured approximately 180 degrees apart from each other on the retina. The superficial and deep vascular networks were distinguished from one another by their z-position within the z-stack. Each z-stack was then collapsed into two MAP’s of the superficial and deep vascular networks. The MAP’s were then regionally thresholded using Otsu’s method and the function *blkproc* (44 μm × 44 μm with 13 μm overlap). We then skeletonized the vessels and performed spur removal. As previously described^53^,^54^, the vascular density was quantified as the length of skeletonized vessel divided by the image area.

### Histological sections

Fellow eyes (non-imaged) were enucleated and fixed as described above. For paraffin section preparation, eyes were submerged in Davidson’s fixative for 24 hours to help preserve retinal morphology, embedded vertically in paraffin along the corneo-scleral axis orientation, and sectioned at 7 μm intervals. Sections were then stained with hematoxylin & eosin (H&E) and imaged at 10× magnification with brightfield microscopy. Sections with the optic nerve were chosen for analysis. Two masked graders (B.T.S. and R.S.) measured retinal layer thicknesses at regions 150 to 200 μm away from the optic nerve (central) and from the peripheral retinal border (peripheral). The NFL/GCL, IPL, INL, OPL, ONL, and PR layers were individually measured using the ImageJ plug-in *distance_between_polylines.java*^55^.

### Statistical analysis

All measurements were first averaged within each animal. A final average was then computed for all animals. Quantitative data was expressed as mean ± standard error of the mean (S.E.M). The statistical test used was an unpaired Student’s t-test (two-tailed with unequal variance). We considered a p-value less than 0.05 to be statistically significant. All analyses were performed using Graphpad Prism (Version 6, GraphPad Software, San Diego California USA).

## Additional Information

**How to cite this article**: Soetikno, B. T. *et al*. Inner retinal oxygen metabolism in the 50/10 oxygen-induced retinopathy model. *Sci. Rep*. **5**, 16752; doi: 10.1038/srep16752 (2015).
